# Diagnostic performance of the Dengue NS1 Ag Rapid Test during early-stage infections at epidemic-affected health care facilities in northeastern and eastern Ethiopia

**DOI:** 10.1016/j.ijregi.2026.100915

**Published:** 2026-05-11

**Authors:** Chalachew Sisay, Netsanet Worku, Tadesse Awoke, Kassahun Alemu

**Affiliations:** 1 Institute of Public Health, College of Medicine and Health Sciences, University of Gondar, Gondar, Ethiopia; 2 Ethiopian Public Health Institute, Addis Ababa, Ethiopia; 3 Department of Human Nutrition, Institute of Public Health, College of Medicine and Health Sciences, University of Gondar, Gondar, Ethiopia; 4 Department of Epidemiology and Biostatistics, Institute of Public Health, College of Medicine and Health Sciences, University of Gondar, Gondar, Ethiopia

**Keywords:** Dengue, Diagnostic performance, NS1, Sensitivity, Predictive value, Ethiopia

## Abstract

**Objectives:**

Early detection of dengue through the non-structural protein 1 (NS1) antigen is critical for timely diagnosis. Although polymerase chain reaction (PCR) remains the reference standard, its cost and technical requirements limit routine use in many epidemic-prone settings. Rapid diagnostic tests (RDTs) provide a practical alternative but require validation against reference assays. This study aimed to evaluate the diagnostic accuracy of the STANDARD Q Dengue NS1 Ag RDT compared with the Trioplex real-time PCR assay for early dengue detection in healthcare facilities in northeastern and eastern Ethiopia between April and August 2024.

**Methods:**

A cross-sectional diagnostic accuracy study was conducted using consecutive sampling of 339 febrile patients aged over 1 year who presented within 5 days of suspected dengue infection. Diagnostic performance was assessed using contingency table analysis, with sensitivity, specificity, predictive values, likelihood ratios, receiver operating characteristic curve analysis, and Cohen κ.

**Results:**

Most participants were urban residents (90%) and aged 20-29 years. The NS1 Ag RDT demonstrated 82.9% overall accuracy, with a sensitivity of 82.6% (95% confidence interval [CI]: 76.3-87.8) and a specificity of 83.2% (95% CI: 76.4-88.7). Positive and negative predictive values were 85.4% (95% CI: 79.3-90.2) and 80.1% (95% CI: 73.1-86.0), respectively. The positive likelihood ratio was 4.92, receiver operating characteristic area was 0.829, and Cohen κ indicated substantial agreement (κ = 0.656). Test positivity peaked on illness days 2 and 3.

**Conclusion:**

The NS1 Ag RDT demonstrated moderate diagnostic accuracy, supporting its application for early-phase dengue detection in epidemic-prone, resource-limited health care facilities where Trioplex reference real-time PCR is not feasible.

## Highlight

• Non-structural protein 1 antigen rapid diagnostic tests (RDTs) showed high sensitivity in early dengue infection, enabling timely diagnosis where molecular assays are unavailable.
• Predictive values of RDTs supported accurate point-of-care decisions, reinforcing their utility in frontline clinical settings.
• Regular evaluation of RDT performance is essential for effective patient management and outbreak confirmation.

## Introduction

Dengue is an acute febrile illness caused by the dengue virus (DENV), which belongs to the family *Flaviviridae* and the genus *Flavivirus*. The four DENV serotypes (1-4) are transmitted to humans primarily by *Aedes aegypti* and *Aedes albopictus* [1,2], driving the world’s fastest-growing mosquito-borne viral disease. In 2024, dengue transmission reached unprecedented global levels, with World Health Organization (WHO) reporting 14.4 million cases, including 7.7 million laboratory-confirmed infections, 52,738 severe cases, 11,201 deaths, and more than 100 affected countries and territories [3,4].

DENV infection can cause a wide range of symptoms, from mild febrile illness to severe disease, including high fever, severe headache, joint and muscle pain, and rash. Recurrent dengue infection is associated with an increased risk of severe disease, which may manifest as hemorrhagic complications, dengue shock syndrome, hospitalization, or death [5].

In recent years, dengue has emerged as a major public health challenge in Ethiopia. Since the first documented outbreak in 2013, the country experienced a major epidemic in 2024, with over 25,000 reported cases and 45 facility-based deaths, placing significant strain on an already overstretched healthcare system [6]. However, the true burden of dengue is likely underestimated in most resource-limited counties because many infections are mild or asymptomatic and limited diagnostic capacity combined with frequent misclassification of mild cases further weaken surveillance, obscuring the disease’s full impact on communities and health services [4,7].

Diagnosing dengue is challenging because its often non-specific symptoms overlap with many other arboviral and non-arboviral infections [4]. Early diagnosis allows timely intervention before complications such as dehydration, plasma leakage, or dengue hemorrhagic fever develop. Prompt detection enables supportive care that can prevent progression to shock and organ failure. Evidence indicates that timely diagnosis and management can reduce mortality in severe cases from over 20% to less than 1%, underscoring the life-saving importance of accessible and reliable diagnostic testing, particularly, in resource-limited health care facilities where dengue burden is frequently underestimated [8].

Despite the clear benefits of early detection, confirming dengue infection remains difficult in many resource-limited health care facilities. Worldwide, diagnosis can be achieved through viral isolation, viral ribonucleic acid (RNA) detection, or serological and molecular methods such as polymerase chain reaction (PCR). However, these techniques are expensive, require specialized equipment and reagents, and depend on trained personnel, all of which limit their availability. Long turnaround times further delay clinical decision-making, leaving many patients without timely confirmation and care [9].

To address these challenges, rapid diagnostic tests (RDTs) have become an important tool for frontline care. The DENV contains a single-stranded RNA genome that encodes three structural and seven non-structural (NS) proteins [10]. Commercially available RDTs can detect NS protein 1 (NS1) antigen (Ag) of DENV in whole blood/serum or plasma [10,11]. The NS1 Ag test demonstrates high sensitivity during the acute stage of infection, beginning as early as the first day after fever onset. Unlike conventional laboratory methods, NS1 Ag RDTs provide results within minutes, are inexpensive, easy to use, and remain stable even at temperatures above 30°C. These features make them particularly valuable in resource-limited health care facilities where timely diagnosis can save lives and reduce the strain on health care systems [12].

In resource-limited health care facilities, RDTs kits play a vital role in strengthening clinical care and public health response. By enabling quick and reliable detection, they support timely treatment decisions, enhance disease surveillance, and guide outbreak investigations. These rapid methods not only improve patient outcomes but also help identify emerging epidemics early, allowing health authorities to implement effective prevention and control measures before the situation escalates [13].

Unfortunately, these diagnostic tests are often used in remote settings where environmental and storage conditions, patient febrile status, and operator performance can affect accuracy. Without proper validation against the reference Trioplex real-time PCR (Trioplex rRT-PCR) assay, the reliability of RDTs remains uncertain. Inaccurate results may lead to misdiagnosis, inappropriate treatment, unnecessary drug use, and delayed outbreak response, ultimately worsening transmission and undermining public health control efforts.

Therefore, this study aimed to evaluate the diagnostic accuracy of the STANDARD Q Dengue NS1 Ag RDT by assessing its sensitivity and specificity against the reference Trioplex rRT-PCR assay among suspected dengue cases during outbreak reported health care facilities in northeastern and eastern Ethiopia. By focusing on early infection, the study seeks to generate context-specific evidence on the reliability of NS1 Ag RDTs, informing clinical decision-making and strengthening dengue surveillance and outbreak response in epidemic-prone, resource-limited health care facilities.

## Methods

### *Study setting, population, and design*

The study was conducted in Dire Dawa City and in the Afar and Somali regions of Ethiopia. These areas share an arid to semi-arid climate, characterized by persistently high temperatures and low, erratic rainfall. In the Afar Region, temperatures frequently exceed 30°C and can rise to as high as 50°C. Annual rainfall across these regions ranges from less than 200 mm to approximately 600 mm, with wet and dry seasons that are highly unreliable and unevenly distributed [14].

This study used an anonymous, cross-sectional design during the epidemic period and prospectively analyzed leftover specimens from dengue suspected patients. A total of 11 health facilities across three epidemic-affected regions were included, with data collection conducted between April and August 2024. Sampling initially began at Mille Health Centre in the Mille district and was subsequently expanded to Logia, Semera, Chifra, Gelalo, Gewane, Awash Arba, and Awash Town health centers in the northeastern Afar Region. As transmission intensified and spread eastward, additional sampling sites were incorporated in Dire Dawa City, specifically, Dil Chora Hospital, Sabian Hospital, and Goro Health Center and in Gode Town through Gode Hospital in the Somali Region.

Eligible patients were individuals aged over 1 year presenting to the outpatient department with acute febrile illness lasting less than 5 days (temperature ≥37.5 °C) and without alternative diagnosis identified by the clinician. Participants were recruited using consecutive sampling, whereby all patients meeting the inclusion criteria during the study period were enrolled. A physician then classified cases as probable or suspected dengue according to the WHO case definition [15], which requires fever plus at least two of the following: nausea or vomiting, rash, headache, retro-orbital pain, myalgia, or arthralgia. Patients admitted to inpatient departments of the health care facilities were excluded to ensure the study focused on early-presenting cases.

### *Sample size*

The sample size for this study was determined using established approaches for diagnostic accuracy research. The calculation aimed to estimate sensitivity and specificity with a precision of ±5% at a 95% confidence level (α = 0.05, Z = 1.96).

The key assumptions were drawn from published literature. A dengue prevalence of 48% was reported in previous literature [16], whereas diagnostic performance estimates from other studies indicated an expected sensitivity of 88% and a specificity of 99% [17]. Separate calculations were performed for sensitivity and specificity using these parameters. The sensitivity estimate required a larger sample size than the specificity estimate, yielding a minimum of 339 participants.

By selecting the higher of the two values, the study ensured adequate statistical power and precision for sensitivity and specificity. Thus, the final required sample size was set at 339 participants, providing sufficient robustness to evaluated diagnostic accuracy of the STANDARD Q Dengue NS1 Ag RDT against the Trioplex rRT-PCR assay during dengue epidemic in Ethiopia.

### *Clinical sample collection and preparation*

All necessary materials were prepared in advance of sample collection, including a 5-cm^3^ syringe, a 5- to 10-mL serum separator tube, sterile gloves, a tourniquet, cotton swabs, 70% alcohol, a test device, a disposable dropper (100 μL), and a waste bin. Based on the clinician’s assessment and the standard case definition, suspected or probable cases were referred to the health facility laboratory. Each patient was assigned a unique identification number, and comprehensive data (age, sex, region, health facility, onset of illness, and date seen at the health facility) were systematically recorded and reported to the principal investigator.

Trained medical laboratory professionals at each health facility collected venous whole blood samples from suspected or probable dengue cases. A volume of 5-10 mL of venous whole blood was collected anonymously for this purpose. Immediately at the point of care, 100 μL of whole blood was dispensed into the specimen well of the STANDARD Q Dengue NS1 Ag RDT kit. The remaining samples were centrifuged at 2000 revolutions per minute for 5 minutes; if a centrifuge was unavailable, samples were maintained at a 45° angle for 60-90 minutes at room temperature. Finally, 2-3 mL of separated serum was transferred into a plain or Nunc tube. These serum samples were then sent to the Ethiopian Public Health Institute (EPHI) Arbovirus Reference Laboratory, in accordance with the national arboviral disease surveillance and response guidelines [18].

In the referring or outbreak-reporting health facilities, serum samples were stored at 2-4°C for no longer than 1 week. During transport, samples were handled in adherence to national biosafety guidelines and shipped in cooled boxes maintained at 2-4°C. Upon arrival, the testing laboratory stored the samples at the same temperature until analysis. After testing, aliquots were prepared for additional investigations or research and preserved at −80°C [18].

### *Index test: STANDARD Q Dengue NS1 Ag RDT principle and procedure*

This RDT kit is a qualitative, membrane-based immunochromatographic assay designed to detect NS1 Ags in human serum, plasma, or whole blood samples. The kits are manufactured by SD Biosensor, Inc., headquartered in Gyeonggi-do, Republic of Korea. The NS1 Ag RDT cassette was used in accordance with the manufacturer’s instructions for use. According to the kit insert, the assay demonstrated a sensitivity of 92.9% and a specificity of 98.7% [19].

The rapid test strip is coated with anti-dengue NS1 antibodies at the test line (T line) and monoclonal anti-chicken immunoglobulin Y antibodies at the control line (C line). At the health care facility, laboratory personnel performed the assay by adding 100 μL of venous whole blood to the specimen well of the test cassette using a disposable dropper. The sample then interacted with monoclonal anti-dengue NS1 antibodies conjugated to gold nanoparticles on the conjugate pad, enabling the detection process.

The sample, together with the monoclonal anti-dengue NS1 gold conjugate, migrated along the membrane by capillary action toward the test line, where it reacted with the immobilized anti-dengue NS1 antibodies. After a migration period of 15-20 minutes, the appearance of two colored lines in the results window (control and test lines) indicated a positive result. The results were interpreted strictly within the manufacturer-recommended timeframe of 15-20 minutes and not beyond.

Each test device was read independently by two blind readers to assess inter-observer agreement. Any discrepancies between the two readers were resolved by a third blind reader whose decision was considered final. Positive and negative laboratory results from the health facilities were recorded by designated laboratory personnel, entered an Excel spreadsheet, and closely supervised by the principal investigator to ensure data accuracy and reliability.

### *Reference standard: Trioplex rRT-PCR assay principle*

In Ethiopia, the Arbovirus Reference Laboratory at the EPHI used the Trioplex rRT-PCR assay to detect and differentiate RNA from dengue, Zika, and chikungunya viruses in human serum samples [18,20]. This assay enables qualitative identification of viral RNA across a variety of clinical specimens. By using specific primers and TaqMan probes, the Trioplex rRT-PCR reliably detected all DENV strains, although it did not provide differentiation among the individual serotypes.

During the process, viral RNA was first converted to complementary deoxyribonucleic acid (DNA). The probes, which were designed to bind to the target DNA, were labeled with fluorescent dyes (FAM (6-carboxyfluorescein) for dengue, HEX (Hexachloro-fluorescein) for chikungunya, and CAL Fluor Red 610 for Zika) and a quencher molecule. Because the Taq polymerase amplified the DNA, it cleaved the probe, separating the fluorescent dye from the quencher. This action caused the fluorescent dye. The resulting increase in fluorescence was detected by the Trioplex rRT-PCR instrument, indicating the presence and quantity of the target viral RNA [20].

### *Statistical analysis*

Descriptive statistics was used to summarize the demographic characteristics of the study participants, including gender, age, region, and the time to presentation (onset of illness). Diagnostic performance of the index and reference tests was evaluated using a single set of patient samples, with sensitivity and specificity calculated from standard contingency tables [21]. Sensitivity was defined as the proportion of true positives among all individuals with the disease, whereas specificity represented the proportion of true negatives among all individuals without the disease [21,22]. Positive predictive value (PPV) was computed as the proportion of positive test results that were true positives and negative predictive value (NPV) as the proportion of negative test results that were true negatives [23]. Positive and negative likelihood ratios (LR+ and LR−) were calculated to assess the test’s ability to rule in and rule out disease, respectively [23,24].

Receiver operating characteristic (ROC) curves were generated by plotting sensitivity against 1–specificity across varying decision thresholds to visualize overall test performance. The area under the ROC curve (AUC) was used as a summary measure of diagnostic accuracy, reflecting the probability that the test correctly distinguishes between individuals with or without disease [25]. Finally, Cohen κ statistic was calculated to evaluate the level of agreement between the index test and the reference diagnostic standard [26].

### *Ethical consideration*

All study procedures were reviewed and approved by the institutional review board (IRB) of the College of Medicine and Health Sciences at the University of Gondar (reference number CMHSSH-UOGIRERC/2/2024). Because the dengue outbreak required a rapid response, written consent forms could not be used. Instead, with IRB approval, the study was explained verbally to each adult participant, and verbal consent was obtained before sample collection.

For participants aged under 18 years, permission was sought from a parent or legal guardian, and the study was explained in simple terms to the child to obtain assent. Participants were informed that leftover samples from routine NS1 diagnostic testing could be used anonymously for research purposes. To ensure confidentiality, all samples were processed without personal identifiers, and laboratory results were not linked to individual identities.

## Results

### *Participants characteristics*

Of the 339 patients suspected of dengue infection, 213 (62.8%) were female and 126 (37.2%) were male. The median age was 27 years (range: 2-68 years). The majority of participants, 305 (90.0%), resided in urban areas. Most patients 254 (75.0%) presented to the health facilities within 3 days of symptom onset (Table 1).

**Table 1:** Characteristics of patients suspected of dengue who presented to health facilities in Afar, Somali, and Dire Dawa Regions during the study period (April-August 2024) (n = 339).

Characteristics	Number (%)
Gender	
Female	213 (62.8%)
Male	126 (37.2%)
Age (years) median (range) = 27 (2-68)	
Residence	
Urban	305 (90.0%)
Rural	34 (10.0%)
Time to presentation	
≤3 days from symptom onset	254 (75.0%)
>3 days from symptom onset	85 (25.0%)
Region	
Afar	201 (59.3%)
Dire Dawa	107 (31.6%)
Somali	31 (9.1%)

The highest proportion of suspected dengue cases occurred among individuals aged 20-29 years (133 cases, 39.2%), followed by those aged 30-39 years (76 cases, 22.4%). Together, these two age groups accounted for more than 60% of all cases, indicating that young adults were the most affected population segment. Adolescents aged 10-19 years contributed 54 cases (15.9%), forming the third largest group. In contrast, children aged 1-9 years and older adults aged ≥60 years accounted for 18 (5.3%) and 10 (3.0%) cases, respectively. Intermediate groups included individuals aged 40-49 years (35 cases, 10.3%) and 50-59 years (13 cases, 3.8%).

STANDARD Q Dengue NS1 Ag RDT identified 178 positive cases, of which 152 were confirmed by the Trioplex rRT-PCR assay, whereas 26 were classified as false positives. Among the NS1 Ag RDT results, 161 were true negatives and 32 were false negatives. Overall, the assay correctly classified 281 cases, corresponding to an agreement of 82.9% with the reference standard. These results indicate that although the NS1 Ag RDT provides rapid and practical case detection in field settings, its diagnostic accuracy is lower than molecular testing, particularly, in terms of false negatives.

### *STANDARD Q Dengue NS1 Ag RDT diagnostic performance*

Among the participants, 184 (54.3%) were confirmed dengue-positive by the Trioplex rRT-PCR reference assay, whereas 178 (52.5%) tested positive using this NS1 Ag test (Table 2). Compared with the reference assay, the NS1 test demonstrated a sensitivity of 82.6% (95% confidence interval [CI]: 76.3-87.8) and a specificity of 83.2% (95% CI: 76.4-88.7).

**Table 2:** Comparison of the STANDARD Q Dengue NS1 Ag Rapid Diagnostic Test with the Trioplex rRT-PCR reference assay for dengue detection in Afar, Somali, and Dire Dawa Regions, April-August 2024 (n = 339).

		Dengue gold-standard Trioplex rRT-PCR		Total
		Positive	Negative	
STANDARD Dengue NS1 Ag Test kit	**Positive**	**152**	**26**	**178**
	**Negative**	**32**	**129**	**161**
		**184**	**155**	**339**

Abbreviation: rRT-PCR, real-time polymerase chain reaction.

The LR+ was 4.92 (95% CI: 3.45-7.04), indicating that individuals with dengue were approximately five times more likely to have a positive index test result than those without dengue. The dengue NS1 RDT demonstrated a PPV of 85.4%, indicating that this proportion of individuals with a positive test result truly had dengue. Conversely, the NPV was 80.1%, indicating that this proportion of individuals with a negative test result did not have dengue (Table 3).

**Table 3:** Diagnostic performance metrics of STANDARD Q Dengue NS1 Ag RDT with confidence interval (CI) in Afar, Somali, and Dire Dawa Regions during the study period (April-August 2024) (n = 339).

Testing performance indicator	Value	(95% CI)
Prevalence	0.543	(0.488-0.597)
Sensitivity	0.826	(0.763-0.878)
Specificity	0.832	(0.764-0.887)
Likelihood ratio (LR+)	4.92	(3.450-7.040)
Positive predictive value	0.854	(0.793-0.902)
Negative predictive value	0.801	(0.731-0.860)

Abbreviations: NS1 Ag, non-structural protein 1 antigen; RDT, rapid diagnostic test.

**Figure 1:**
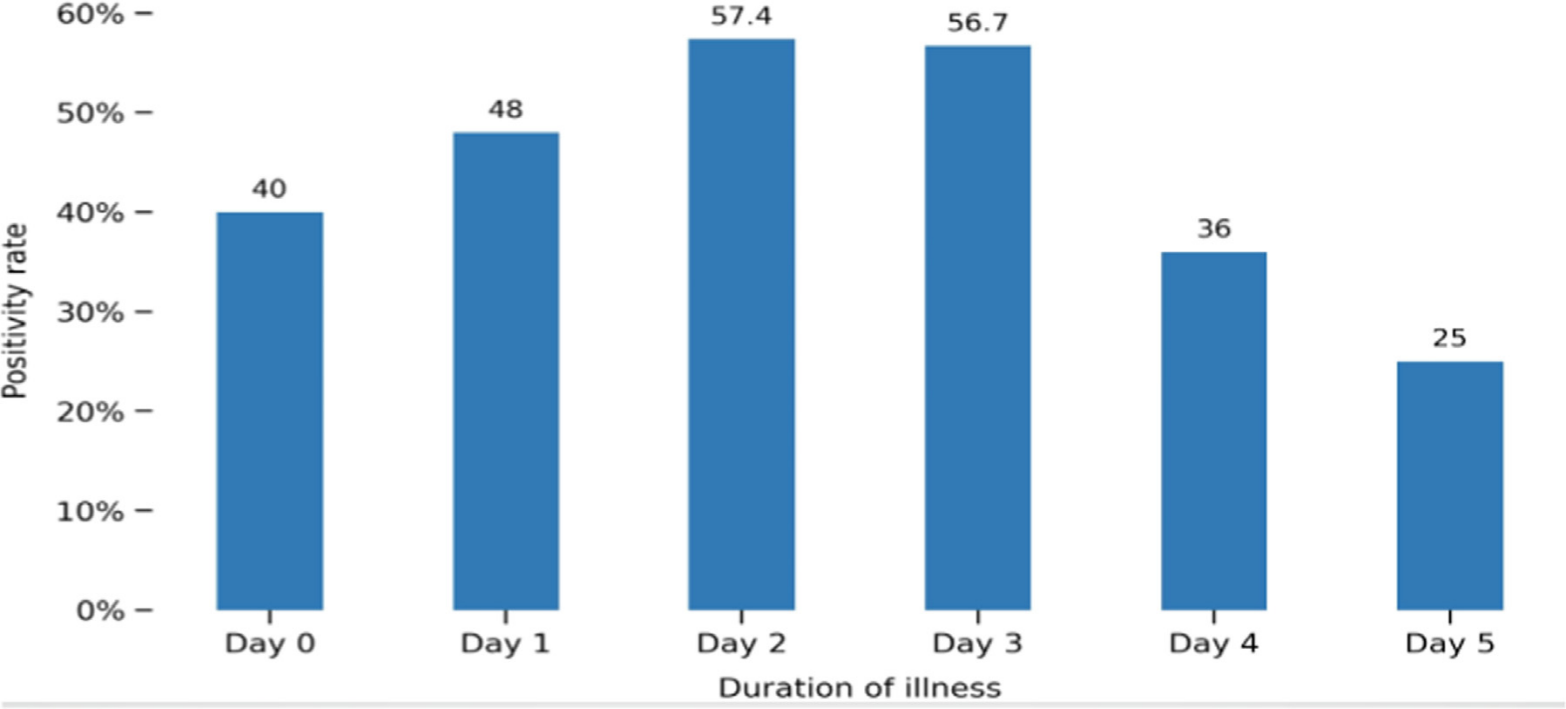
Dengue STANDARD Q Dengue NS1 Ag RDT positivity rate by duration of illness in Afar, Somali, and Dire Dawa Regions during the study period (April-August 2024) (n = 339). Abbreviations: NS1 Ag, non-structural protein 1 antigen; RDT, rapid diagnostic test.

Cohen κ statistics indicated substantial agreement (κ = 0.656, standard error = 0.05, z = 12.09, *P* < 0.001) between the index and reference assay, confirming the agreement of the two paired tests. Figure 1 shows that the dengue NS1 Ag RDT performed optimally on days 2 and 3 of illness, with positivity rates of 57.4% and 56.7%, respectively. During the early phase of illness, on days 0 and 1, the test demonstrated moderate sensitivity, detecting only 40-48% of cases, respectively.

The cycle threshold (Ct) values for Triplex rRT-PCR showed a wide range of viral loads within the tested individuals, from very high (Ct = 18) to very low (Ct = 35). The range of 17 cycles (from 18 to 35) is quite significant, and the average and median values (around 27-28) suggest that the typical viral load is moderate.

### *Performance evaluation using ROC curve*

**Figure 2:**
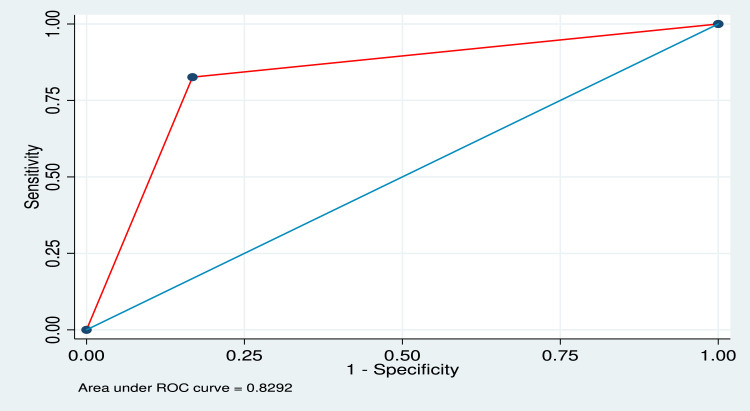
Area under the receiver operating characteristic (ROC) curve illustrating the diagnostic performance of the STANDARD Q Dengue NS1 Ag Rapid Test kit in Afar, Somali, and Dire Dawa Regions, April-August 2024 (n = 339) (sensitivity and 1–specificity) are plotted; area under the ROC curve = 0.829. Abbreviation: NS1 Ag, non-structural protein 1 antigen.

The diagnostic performance of the NS1 Ag RDT was evaluated against Trioplex RT-PCR assay as the reference standard. The ROC curve for the index test lay well above the diagonal line of no discrimination, indicating performance substantially better than random chance. The AUC was 0.829 (95% CI: 0.790-0.870), demonstrating good overall accuracy. Because values above 0.80 are generally considered strong, this result suggests that the index test has a high probability of correctly classifying patients who were dengue NS1–positive and -negative (Figure 2).

## Discussion

This study demonstrates that the STANDARD Q Dengue NS1 Ag RDT shows good diagnostic performance compared with the Trioplex rRT-PCR assay, the established reference standard. The observed sensitivity and specificity of over 82%, together with substantial agreement between the two assays (κ = 0.656) and strong discriminatory ability reflected by the ROC analysis (AUC = 0.829), support the overall reliability of this NS1 Ag RDT for dengue detection. These findings indicate that the test performs well in identifying dengue cases during outbreak conditions.

This NS1 Ag RDT assay was particularly effective during the early phase of illness, especially on days 2 and 3 after symptom onset. This early diagnostic utility reinforces the value of NS1 Ag RDT as frontline tools for timely clinical decision-making, enabling prompt patient management and risk stratification before severe complications develop. Early identification of cases is also critical for initiating public health interventions to limit onward transmission.

Our findings are consistent with previous evaluations of NS1-based RDTs, which have demonstrated favorable performance during the acute phase of dengue infection [27,28]. When considered alongside the diagnostic accuracy and early-phase utility observed in this study, these results further support the integration of NS1 Ag–based RDT into routine clinical care and dengue surveillance programs. Their use can help bridge diagnostic gaps, particularly, in areas where access to molecular testing is limited [5].

Importantly, the ability of NS1 Ag RDT to provide reliable results early in the course of illness has meaningful implications for outbreak control, clinical case management, and broader public health response. In resource-limited and outbreak-prone health care facilities, where laboratory infrastructure and molecular diagnostics may be constrained, rapid point-of-care testing can substantially improve the timeliness of diagnosis and intervention. Strengthening the use of validated NS1 Ag RDT diagnostics, alongside context-specific performance evaluation, may, therefore, enhance outbreak detection, case management, and surveillance capacity in dengue-affected areas of the country.

The NS1 Ag RDT evaluated in this study demonstrated a sensitivity of 82.6% and a specificity of 83.2%. Compared with an Indonesian study that assessed five similar assays, our results showed higher sensitivity but did not reach the 100% specificity reported in that setting [29]. However, our findings are more consistent with multicenter evaluations conducted in Peru, which reported sensitivities ranging from 71.9% to 79.1% [30]. Collectively, these results suggest that although the NS1 Ag RDT may not match the specificity of some leading assays, it nonetheless represents a reliable and practical diagnostic option for clinicians, particularly, in resource-limited and outbreak-prone health care facilities.

The clinical utility of the NS1 Ag–based RDT in this study is highlighted by its PPV of 85.4% and NPV of 80.1%. In the context of a dengue prevalence of 54.3%, a positive result corresponds to an 85.4% probability of true infection, thereby justifying immediate clinical management and timely public health interventions. These findings are consistent with a multicenter evaluation of six NS1 RDTs conducted in Singapore, which reported PPVs ranging from 82% to 88% and NPVs between 78% and 83% [31]. Similarly, a study validating the Bioline Dengue Duo rapid test in Myanmar found PPVs of 84% and NPVs of 79%, supporting the reliability of NS1 Ag RDT assays in endemic areas [32]. our results underscore the robustness of the STANDARD Q NS1 Ag RDT as a viable diagnostic option.

Conversely, the NPV of 80.1% indicates a substantial 20% residual risk of dengue infection after a negative NS1 Ag–based RDT result. This finding underscore that a negative rapid test alone is insufficient to definitively exclude dengue, particularly, in endemic areas of the country. For frontline health care workers, this means that patients with strong clinical suspicion should not be discharged solely on the basis of a negative NS1 result. Instead, confirmatory testing with Trioplex rRT-PCR assay or serological assays should be pursued.

In resource-limited health care facilities, close clinical monitoring, repeat testing after 48-72 hours, and timely referral to higher-level facilities are recommended strategies to mitigate the risk of missed diagnoses [33], which underscores the risk of false negatives when relying solely on NS1 Ag RDT. These measures highlight the importance of confirmatory assays and vigilant clinical follow-up in epidemic-prone areas. It is important to note that predictive values vary with disease prevalence; although the high PPV supports the test’s use for confirmation during outbreaks, in lower-prevalence settings, the PPV would decrease and the NPV would increase, shifting the test’s utility toward ruling out infection [34].

The diagnostic performance of NS1 Ag RDT was significantly influenced by the timing of sample collection after the onset of illness [33]. In this study, the NS1 Ag RDT showed suboptimal sensitivity on the first day of illness but improved performance on days 2 and 3, with positivity rates of 57.4% and 56.7%, respectively. This temporal pattern is consistent with previous evaluations of NS1 assays, which report peak sensitivity between days 2 and 4 after symptom onset and lower detection at the very beginning or later in the clinical course [30]. Other studies comparing NS1 platforms have also shown increasing sensitivity from day 0 to day 3 after fever onset [31].

The ROC analysis in this study demonstrated that the NS1 Ag RDT had good overall diagnostic performance, with an AUC of 0.829. indicating that the assay reliably distinguishes individuals who are dengue-positive from those who are dengue-negative. An AUC in this range is generally interpreted as showing good accuracy for a diagnostic test and supports the use of this RDT as a dependable tool for dengue diagnosis. These findings are aligned with previous evaluations of NS1-based assays, which have reported AUC values in the “good to excellent” range and have highlighted the clinical value of NS1 testing for early detection of dengue infection in patients with acute-phase illness [35].

The agreement between the NS1 Ag RDT kit and the Trioplex rRT-PCR assay was substantial, with a Cohen κ value of 0.656. According to established interpretation criteria, κ values between 0.61 and 0.80 indicate substantial agreement, supporting the reliability of the NS1 assay in clinical settings [28]. These findings are consistent with previous studies. For instance, a multicenter evaluation in Singapore reported κ values ranging from 0.60 to 0.81 across several NS1 Ag–based RDT, highlighting their consistent performance across diverse epidemiologic context [36]. The examined kit showed a substantial agreement, suggesting that the NS1 Ag RDT represents a reliable tool for the detection of dengue, particularly, in resource-limited health care facilities.

RDTs, which detect DENV NS1 Ag, provide a practical solution in health care facilities lacking comprehensive laboratory service. This test offers the ability to confirm dengue infection during outbreaks of acute febrile illness, where advanced molecular diagnostic methods might be unavailable. For example, a study in Puntland State, Somalia demonstrated the efficacy of these tests in identifying dengue cases, with most confirmed cases showing positive results for NS1 Ag [37].

This study has several limitations. The evaluation of the STANDARD Q Dengue NS1 Ag RDT kit was conducted solely compared with the Trioplex rRT-PCR assay, without parallel testing against other commercial kits. Sample collection was uneven across the three geographical regions, and key environmental factors such as local temperature were not documented, limiting insights into their potential impact on kit performance. Circulating DENV serotypes were not assessed, despite their known influence on diagnostic sensitivity and clinical management; thus, health care providers in endemic regions should consider local serotype prevalence when interpreting results because NS1 Ag–based rapid tests are most reliable within the first 5 days of fever onset.

The diagnostic accuracy in this study was limited by reliance on a single NS1 Ag test format, which produced false-positive and false-negative rates of 16.8% and 17.4%, respectively. These inaccuracies may have arisen from cross-reactivity with other *Flaviviridae* viruses, secondary infections in individuals with preexisting antibodies, or low viral loads during the early or late stages of disease. In addition to these virological factors, the Ethiopian context presents further challenges because malaria and other endemic febrile illnesses share overlapping clinical features with dengue. Such co-endemic conditions may influence the perceived specificity of the RDT and complicate its practical application in routine outbreak investigations.

To improve diagnostic reliability and reduce misclassification, future research should explore multi-Ag panels or incorporate serotype-specific controls approach. These strategies would enhance diagnostic accuracy, mitigate the risk of misdiagnosis in areas where malaria and dengue co-exist, and, ultimately, strengthen evidence-based outbreak responses in Ethiopia’s dengue-prone areas.

## Conclusion

The STANDARD Q Dengue NS1 Ag RDT kit demonstrated moderate diagnostic accuracy for early dengue detection, particularly, among patients presenting on days 2 and 3 of illness. Sensitivity, specificity, ROC curve analysis, and Cohen κ statistics all supported its role as a practical rapid confirmatory tool, showing substantial agreement with the reference standard.

Although less robust than the Trioplex rRT-PCR assay largely due to its false-negative rate, the RDT offers meaningful utility in ruling out infection during the early stage of disease. When applied with an understanding of its performance characteristics, this test can enhance patient triage, support timely clinical management, and strengthen outbreak response. Its ease of use and applicability in resource-limited, dengue epidemic-prone health care facilities highlight its potential contribution to clinical care and public health strategies.

## Declaration of competing interest

The authors have no competing interests to declare.
